# Geneva Medical College

**Published:** 1848-11

**Authors:** 


*Geneva Medical College.*—The annual lecture term in this institution commenced on the first Tuesday in October. We learn that the class in attendance numbers 52. The lectures on the Institutes and Practice of Medicine are to be given by Prof. Wm. Sweetser, of the College at Castleton, Vt., appointed *vice* the editor of this Journal, who declined the appointment.

